# Supplementary material for: Resolving the full spectrum of human genome variation using Linked-Reads
Source: Genome Res. 2019 Apr;29(4):635–45. doi: 10.1101/gr.234443.118 (PMC6442396; doi:10.1101/gr.234443.118)
Supplement: Supplemental Material [file supp_gr.234443.118_Supplemental_longranger-2.2.zip › longranger-2.2.tar.bz2!/lr2.2-public/lib/stan/cmdstan/stan/doc/api/html/write__iteration_8hpp_source.html]

Stan: src/stan/common/write\_iteration.hpp Source File


|  |  |
| --- | --- |
|  | Stan  2.5.0  probability, sampling & optimization |


- Main Page
- Related Pages
- Namespaces
- Classes
- Files

- File List
- File Members

All Classes Namespaces Files Functions Variables Typedefs Enumerations Enumerator Friends Macros Pages

- src
- stan
- common

write\_iteration.hpp

Go to the documentation of this file.

1 #ifndef STAN\_\_COMMON\_\_WRITE\_ITERATION\_HPP

2 #define STAN\_\_COMMON\_\_WRITE\_ITERATION\_HPP

3

4 #include <ostream>

5 #include <vector>

6 #include <stan/common/write\_iteration\_csv.hpp>

7

8 // FIXME: write\_iteration calls std::cout directly.

9 // once removed, remove this include

10 #include <iostream>

11

12

13 namespace stan {

14

15  namespace common {

16

17

18  template <class Model, class RNG>

19  void write\_iteration(std::ostream& output\_stream,

20  Model& model,

21  RNG& base\_rng,

22  double lp,

23  std::vector<double>& cont\_vector,

24  std::vector<int>& disc\_vector) {

25  std::vector<double> model\_values;

26  model.write\_array(base\_rng, cont\_vector, disc\_vector, model\_values,

27  true, true, &std::cout);

28  write\_iteration\_csv(output\_stream, lp, model\_values);

29  }

30

31  } // namespace common

32

33 } // namespace stan

34

35 #endif

write\_iteration\_csv.hpp

stan::common::write\_iteration

void write\_iteration(std::ostream &output\_stream, Model &model, RNG &base\_rng, double lp, std::vector< double > &cont\_vector, std::vector< int > &disc\_vector)

**Definition:** write\_iteration.hpp:19

stan::common::write\_iteration\_csv

void write\_iteration\_csv(std::ostream &output\_stream, const double lp, const std::vector< double > &model\_values)

**Definition:** write\_iteration\_csv.hpp:10


---

[ Stan Home Page ]

*© 2011–2014,
Stan Development Team.*
